# Ferulic Acid Ameliorates Atherosclerotic Injury by Modulating Gut Microbiota and Lipid Metabolism

**DOI:** 10.3389/fphar.2021.621339

**Published:** 2021-03-25

**Authors:** Yuyan Gu, Yaxin Zhang, Mei Li, Zhiyong Huang, Jing Jiang, Yihao Chen, Junqi Chen, Yuhua Jia, Lihua Zhang, Fenghua Zhou

**Affiliations:** ^1^ School of Traditional Chinese Medicine, Southern Medical University, Guangzhou, China; ^2^ VIP Healthcare Center, Third Affiliated Hospital of Sun Yat-sen University, Guangzhou, China; ^3^ Department of Otolaryngology, The Third Affiliated Hospital of Southern Medical University, Guangzhou, China; ^4^ Department of Gynaecology, Integrated Hospital of Traditional Chinese Medicine, Southern Medical University, Guangzhou, China

**Keywords:** atherosclerosis, lipid metabolism, gut microbiota, metabolites, ferulic acid

## Abstract

Atherosclerosis is a leading cause of death worldwide. Recent studies have emphasized the significance of gut microbiota and lipid metabolism in the development of atherosclerosis. Herein, the effects and molecular mechanisms involving ferulic acid (FA) was examined in atherosclerosis using the ApoE-knockout (ApoE^-∕-^, c57BL/6 background) mouse model. Eighteen male ApoE^−/−^ mice were fed a high-fat diet (HFD) for 12 weeks and then randomly divided into three groups: the model group, the FA (40 mg/kg/day) group and simvastatin (5 mg/kg/day) group. As results, FA could significantly alleviate atherosclerosis and regulate lipid levels in mice. Liver injury and hepatocyte steatosis induced by HFD were also mitigated by FA. FA improved lipid metabolism involving up-regulation of AMPKα phosphorylation and down-regulation of SREBP1 and ACC1 expression. Furthermore, FA induced marked structural changes in the gut microbiota and fecal metabolites and specifically reduced the relative abundance of *Fimicutes*, *Erysipelotrichaceae* and *Ileibacterium,* which were positively correlated with serum lipid levels in atherosclerosis mice. In conclusion, we demonstrate that FA could significantly ameliorate atherosclerotic injury, which may be partly by modulating gut microbiota and lipid metabolism via the AMPKα/SREBP1/ACC1 pathway.

## Introduction

Cardiovascular and cerebrovascular diseases, which mainly develop from atherosclerosis, have become the leading cause of death worldwide, especially in developed countries (Liu et al., 2019). Atherosclerosis is characterized by excessive cholesterol deposition within the intima, especially in the aorta and coronary artery. A lipid metabolism disorder has been considered the key pathological mechanism involved in the pathogenesis of atherosclerosis.

Cholesterol has been considered the primary promoter of atherosclerosis development for nearly a century. The atherosclerotic plaque, derived from passive lipid accumulation within the artery wall, is the most significant pathological change of atherosclerosis visible under the microscope (Luo et al., 2015). AMP-activated protein kinase (AMPK), a key regulator of lipid and energy metabolism, can improve lipid metabolism, including lipogenesis, lipolysis, lipid transport, and lipid oxidation (Ma et al., 2017; Park et al., 2017). AMPK not only reduces lipogenesis by activating sterol regulatory element binding protein 1 (SREBP1) and acetyl-CoA carboxylase (ACC) (Jung et al., 2012; Park et al., 2017), but also regulates lipid transport by mediating reverse cholesterol transport (Ma et al., 2017). Furthermore, AMPK modulates lipid oxidation through the AMPK/proliferator-activated receptor-γ coactivator 1α/peroxisome proliferator-activated receptor α (PPARα) pathway (Araújo et al., 2020).

The gut microbiota is an important part of the gut microenvironment. Recently, a growing body of evidence has revealed that the gut microbiota is closely associated with the development of atherosclerosis (Tang et al., 2019). The gut microbiota and its products can regulate lipid metabolism and the immune system, which play key roles during the progression of atherosclerosis (Jonsson and Bäckhed, 2017). Oral administration of probiotics, such as *Lactobacillus,* not only prevents atherosclerosis, but also regulates the expression of AMPK, SREBP1, ACC, ATP-binding cassette transporter A1 (ABCA1), ATP-binding cassette transporter G1, peroxisome proliferator-activated receptors, and liver X receptor (LXR), factors that associate with lipid metabolism in mice fed a high-fat diet (HFD) (Zhao et al., 2019). Furthermore, the products of the gut microbiota, such as short chain fatty acids (SCFAs) and amino acids, absorbed from bowel and transported to the liver, can modulate energy homeostasis and lipid metabolism (Araújo et al., 2020; Deroover et al., 2017; Zhang et al., 2007). Taken together, the gut microbiota may exert an important role in the development of atherosclerosis, although the mechanism still remains unclear.

Ferulic acid (FA) belongs to the family of phenolic acids and is abundant in *Angelica sinensis (Oliv.) Diels* and *Ligusticum chuanxiong.* Previous studies have reported that FA relieved atherosclerosis through antioxidant, anti-hyperlipidemic, and anti-inflammatory effects (Chmielowski et al., 2017). However, the exact pharmacological mechanisms involved remain to be clarified. Recently, researchers have shown that FA modulates the gut microbiota composition in mice presenting nonalcoholic fatty liver disease and increased levels of intestinal *Lactobacillus* in the transverse aortic constriction mouse model (Liu et al., 2019; Ma et al., 2019). Nonetheless, whether FA could regulate gut microbiota composition and metabolism in atherosclerosis has not been reported. Therefore, we aimed to investigate the effects of FA on the gut microbiota and lipid metabolism in the atherosclerotic mice and clarify its molecular mechanism.

## Materials and Methods

### Materials and Reagents

FA (purity >98.00%) was purchased from Chengdu Mansite Biotechnology Co., Ltd. (China)., and simvastatin tablets from Hangzhou MSD Pharmaceutical Co., Ltd. (China). Antibodies against phosphorylated-AMPKα/AMPKα, ACC1, GAPDH, and goat anti-rabbit antibodies were purchased from Affinity Biosciences Ltd (USA). Antibodies against SREBP1 were obtained from Beijing Bioss Biotechnology Co., Ltd (China), and DyLight 488/549, goat anti-rabbit lgG was from Abbkine Scientific Co., Ltd. (United States). Assay kits for Masson trichrome and Oil Red-O (ORO) staining, triglycerides (TG), and reagent kits for assessing total cholesterol (TC), low density lipoprotein-cholesterol (LDL-C), and high density lipoprotein-cholesterol (HDL-C) were purchased from NanJing JianCheng Bioengineering Institute (China). SYBR Green qPCR Supermix, PrimeScript™ RT reagent Kit with gDNA Eraser were purchased from Takara Bio Inc. (Japan). GoTaq^®^ qPCR Master Mix was obtained from Vazyme-innovation in enzyme technology (China), primers for GAPDH, ABCA1, LXRα, SREBP1, AMPK, ACC1, fatty acid synthase (FASN) and stearoyl-CoA desaturase-1 (SCD1) were from Shanghai Sangon Biotech Co., Ltd. (China).

### Animal Models

The following experiments were approved by the Animal Care and Use Committee of Southern Medical University. Eighteen eight-week-old male ApoE^−/−^ (c57BL/6 background) and six eight-week-old male c57BL/6 mice were purchased from Vital River Laboratories Co., Ltd., which distributed originally from The Jackson Laboratory. ApoE^−/−^ mice were fed with a HFD (21% fat +0.15% cholesterol), while c57BL/6 mice were fed a common diet. After 12 weeks, 18 ApoE^−/−^ mice were randomly divided into groups treated as follows: the model group (*n* = 6), the FA group (40 mg/kg/day) (*n* = 6), the simvastatin group (5 mg/kg/day) (*n* = 6), and six c57BL/6 mice served as the control group. FA and simvastatin were administered by gavage 5 days per week for 12 weeks. The mice in the control and model groups were given an equal volume of saline. All mice were housed in a controlled environment (22 ± 2°C, in 55 ± 5% relative humidity, with a 12-h light/dark cycle) and given water and food ad libitum. Mice were weighed every 4 weeks.

### Serum Biochemical Analysis

Mice were anesthetized with chloral hydrate (10 mg/kg) and blood samples were collected by cardiac puncture after fasting for 12 h. Serum levels of SOD, MDA, LDH, AST, ALT, TC, TG, LDL-C, and HDL-C were measured using the respective assay kits following the manufacturer’s instructions.

### Tissue Staining

The heart, aorta, and liver of mice were collected and histological sections were prepared. Frozen sections of the aortic root and liver were subjected to ORO staining and Masson trichrome staining following the protocols indicated by the manufacturers. Paraffin-embedded sections of aortic and liver tissue slices were stained with hematoxylin and eosin (H&E).

### Quantitative Real-Time PCR

Total RNA was extracted from the mice liver, and cDNA was synthesized using PrimeScript™ RT reagent Kit according to the manufacturer’s protocol. GAPDH was used as an internal control. The primer sequences of the target genes are shown in Table 1. The reaction conditions were as follows: 95°C pre-denaturation for 10 min; 95°C denaturation for 10 s, annealing at 60°C for 15 s, for a total of 40 cycles, followed by melting curve analysis, 95°C for 15 s, 60°C for 15 s, and 95°C for 15 s. The mRNA levels were calculated using the 2^−ΔΔ^ CT method.

**TABLE 1:** Primer sequences for quantitative real-time PCR amplification.

Gene name	Gene number	Sequence 5′–3′
GAPDH	XM_036165840.1	Forward: 5′-AGG​TCG​GTG​TGA​ACG​GAT​TTG-3′
		Reverse: 5′-TGT​AGA​CCA​TGT​AGT​TGA​GGT​CA-3′
AMPK	NM_001384157.1	Forward: 5′-GAG​GTT​CAC​AGT​GCC​CTT​CT-3′
		Reverse: 5′-TGG​GGT​TTC​ATT​GGA​CTG​CT-3′
SREBP1	XM_036156491.1	Forward: 5′-GCA​GCC​ACC​ATC​TAG​CCT​G-3′
		Reverse: 5′-CAG​CAG​TGA​GTC​TGC​CTT​GAT-3′
ACC1	XM_036156218.1	Forward: 5′-ATG​GGC​GGA​ATG​GTC​TCT​TTC-3′
		Reverse: 5′-TGG​GGA​CCT​TGT​CTT​CAT​CAT-3′
LXRα	XM_006499168.4	Forward: 5′-CTC​AAT​GCC​TGA​TGT​TTC​TCC​T-3′
		Reverse: 5′-TCC​AAC​CCT​ATC​CCT​AAA​GCA​A-3′
ABCA1	XM_006537554.2	Forward: 5′-GCT​TGT​TGG​CCT​CAG​TTA​AGG-3′
		Reverse: 5′-GTA​GCT​CAG​GCG​TAC​AGA​GAT-3′
SCD1	NM_009127.4	Forward: 5′-TTC​TTG​CGA​TAC​ACT​CTG​GTG​C-3′
		Reverse: 5′-CGG​GAT​TGA​ATG​TTC​TTG​TCG​T-3′
FASN	XM_030245556.1	Forward: 5′-GGA​GGT​GGT​GAT​AGC​CGG​TAT-3′
		Reverse: 5′-TGG​GTA​ATC​CAT​AGA​GCC​CAG-3′

### Western Blotting

Liver protein was quantified using the BCA method. Total protein (60 μg) was resolved by 10% SDS-PAGE and transferred to PVDF membranes. After blocking at room temperature (RT) for 2 h in 0.5% BSA, membranes were incubated with primary antibody at 1:500 overnight at 4°C, and then washed with TBST 3 times. Membranes were incubated with secondary antibody at 1:3,000 for 2 h at RT. Protein bands were detected by ECL and analyzed using Image J software.

### Immunofluorescence

Frozen sections were fixed with 4% paraformaldehyde for 30 min at RT. After permeating with 0.3% Triton X-100 for 10 min, sections were blocked with 10% goat serum for 1 h at RT and incubated with primary antibodies (1:250) overnight at 4°C. Sections were incubated with secondary antibody (1:400) in the dark for 2 h at RT, following staining of nuclei with DAPI for 10 min. Sections were then photographed by fluorescence microscopy.

### Gut Microbiota Analysis

At the end of 12 weeks of treatment, fresh fecal samples (5–8 pellets/mouse) were collected one day before the sacrifice of mice and stored at −80°C. Total genomic DNA was extracted using MOBIO PowerSoil^®^ DNA Isolation Kit (MOBIO Laboratories, Carlsbad, CA, United States). The V3–V4 region of 16S rRNA for each species was amplified using forward primer 338F (5′-GGACTACHVGGGTWTCTAAT-3′) and reverse primer 806R (5′-ACT​CCT​ACG​GGA​GGC​AGC​A-3′). PCR reactions, containing 25 μl 2x Premix Taq, 1 μl each primer (10 mM) and 3 μl DNA (20 ng/μl) template in a volume of 50 µl, were amplified by thermocycling: 5 min at 94°C for initialization; 30 cycles of 30 s denaturation at 94°C, 30 s annealing at 52°C, and 30 s extension at 72°C; followed by 10 min final elongation at 72°C. After detecting by 1% agarose gel electrophoresis, PCR products were mixed in equidensity ratios according to the GeneTools Analysis Software (Version4.03.05.0, SynGene). Then, mixture PCR products were purified with EZNA Gel Extraction Kit (Omega, United States). Sequencing libraries were generated using NEBNext^®^ Ultra™ DNA Library Prep Kit for Illumina^®^ (New England Biolabs, MA, United States). The library quality was assessed on the Qubit@ 2.0 Fluorometer (Thermo Fisher Scientific, MA, United States) and Agilent Bioanalyzer 2100 system (Agilent Technologies, Waldbron, Germany). At last, Libraries were sequenced on an IlluminaHiseq 2500 platform (Guangdong Magigene Biotechnology Co., Ltd. Guangzhou, China). Sequencing data were uploaded to NCBI database, and the accession number is PRJNA678598.

The paired-end raw reads were quality filtered using Trimmomatic (V0.33). Paired-end clean reads were merged using FLASH (V1.2.11). Sequences were assigned to each sample based on their unique barcode and primer using Mothur software (V1.35.1). According to usearch software (V10), sequences with ≥97% similarity were assigned to the same operational taxonomic units (OTUs) (Fan et al., 2019). After singleton OTU, chimera, and contamination OTU removal, OTUs were normalized. For each representative sequence, the silva (https://www.arb-silva.de/) database was used to annotate taxonomic information (set the confidence threshold to default to ≥0.5). Next, QIIME (V1.9.1) was used to calculated Alpha (*α*) diversity including the value of Chao1, Shannon and Simpson. Beta (*β*) diversity was evaluated by principal coordinate analysis (PCoA) on unweighted UniFrac distance matrix. Non-parametric multivariate analysis of variance (adonis) using distance matrices were performed by R software.

### Fecal Metabolomic Analysis

Fecal samples were collected and the supernatants were loaded onto a nuclear magnetic resonance (NMR) tube and sealed using a 3 KDa Millipore Amicon^®^ ULTRA. NMR spectroscopy data were collected on a Bruker AV III 600 MHz spectrometer under the following conditions: temperature (K): 298.03, NMR frequency (MHz): 600.20, transients (scans): 128, frequency domain size: 131,072, spectral width: 8,403.361, time domain size: 65,536, and pulse sequence: noesygppr1d. Next, data was normalized using Pareto scaling and analyzed using partial least square-discriminant analysis (PLS-DA). Variable Importance in Projection (VIP) scores were used to select differential metabolites between groups. Candidate metabolites having VIP > 1 and *p*  <  0.05 were selected as potential biomarkers.

### Statistical Analysis

Differential levels of serum biochemical variables, plaque area, mRNA, protein, the relative abundance of gut microbiota and the segmented integration of metabolites were analyzed using graphpad prsim 8 (GraphPad Software, Inc. USA). Part of the 16S rRNA analysis was carried out in R software. Spearman correlation was analyzed using software SPSS 22.0 (IBM SPSS Statistics, IL, USA). All data was expressed as mean ± S.E.M. One-way ANOVA and T-test were used when data accorded with normal distribution and homogeneity of variance. Welch’s ANOVA and Welch-corrected T-test were used when data accorded with normal distribution but no homogeneity of variance. A *p*-value < 0.05 indicated that the difference was statistically significant.

## Results

### FA Regulated Lipid Levels in HFD-fed ApoE^−/−^ Mice

**FIGURE 1:**
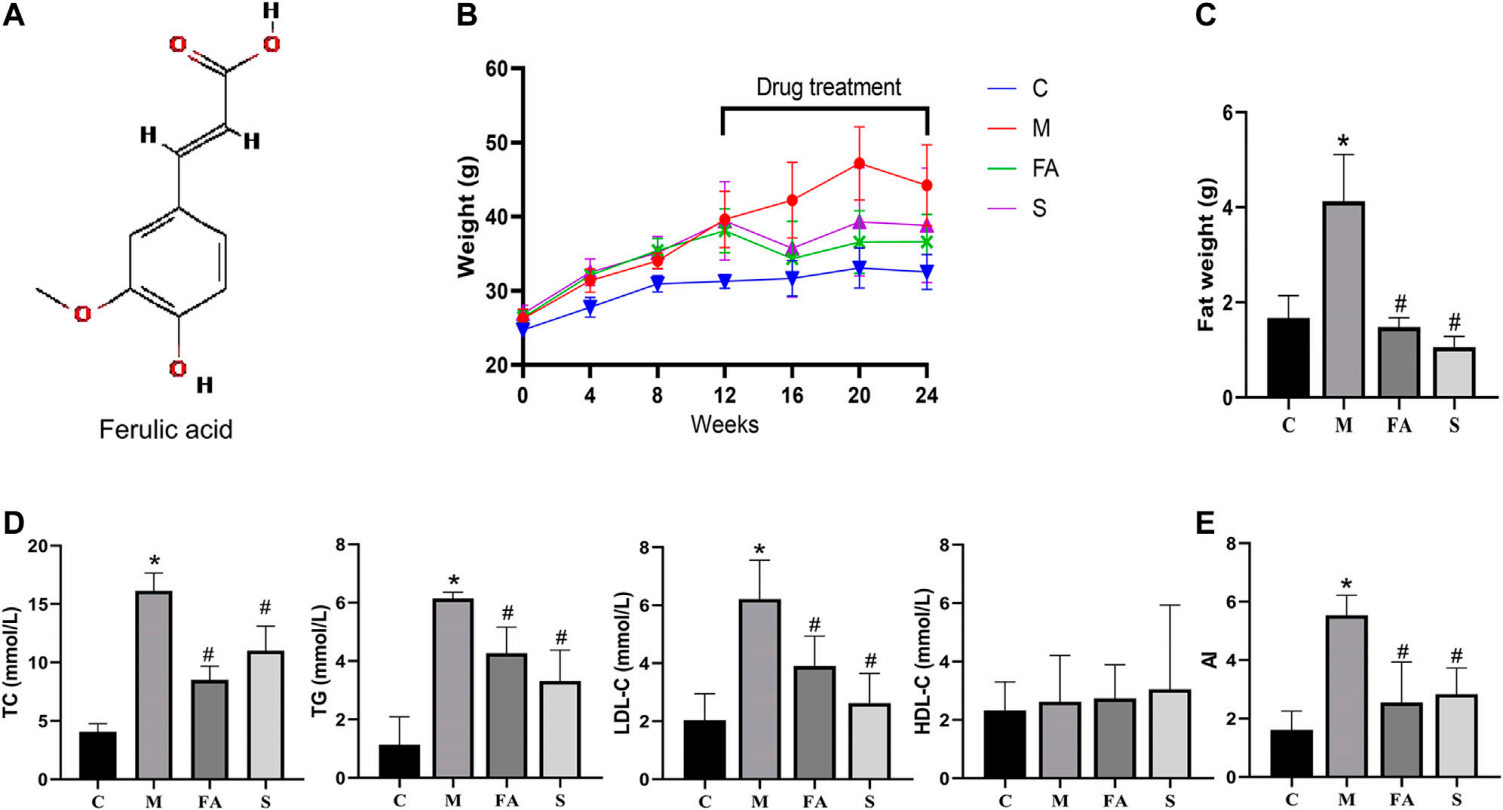
FA regulated lipid levels in HFD-fed ApoE^−/−^ mice **(A)** Chemical structure of FA. **(B)** The change of body weight. **(C)** Abdominal adipose weight of mice. **(D)** Levels of serum lipids (TC, TG, LDL-C, HDL-C) in per group. **(E)** Atherogenic index of various groups. Control group (C), model group (M), Ferulic acid treatment group (FA), simvastatin treatment group (S). *n* = 4. *p < 0.05, **p < 0.01; as compared to the control group. ^#^ *p* < 0.05; as compared to the model group.

The chemical structure of FA is shown in Figure 1A. Firstly, we found that FA administration resulted in less body weight gain between the 16th and 20th week (Figure 1B; *p* < 0.05). In Figure 1D, there was a significant increase in TC, TG, and LDL-C levels in model mice compared to control mice. However, the HDL-C level showed no significant differences in the model and control group. Specifically, both FA and simvastatin markedly decreased levels of TC, TG, and LDL-C (*p* < 0.05). Aherogenic index (AI) was used to evaluate the risk of developing cardiovascular diseases (Sasso et al., 2019). We found that the AI was significantly higher in the model group than that in the control group, while FA could significantly reduce the AI (2.17-fold; *p* < 0.05; Figure 1E). Results were similarly in the simvastatin group. In summary, the result suggests that FA could greatly regulate lipid levels in ApoE^−/−^ mice.

### FA Reduced Atherosclerosis Injury in ApoE^−/−^ Mice

**FIGURE 2:**
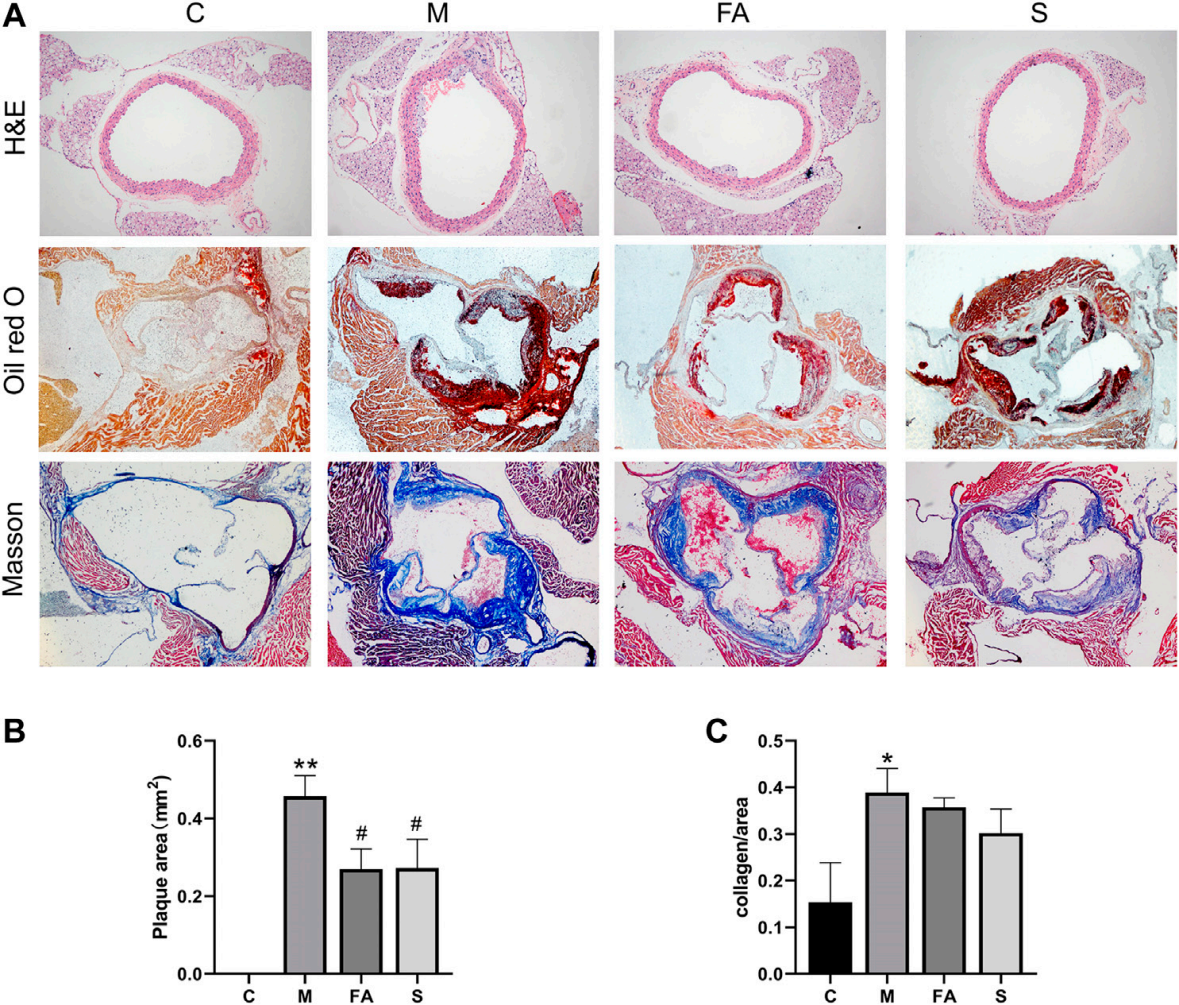
FA reduced atherosclerosis injury in ApoE^−/−^ mice **(A)** H&E staining of aorta (100×), Oil Red O and Masson staining of aortic root (40×). **(B)** Quantitative analysis of plaque area in aortic root (*n* = 3). **(C)** Quantitative analysis of collagen fibers in aortic root (*n* = 3). *p < 0.05, **p < 0.01; as compared to the control group. ^#^ *p* < 0.05; as compared to the model group.

To evaluate the effects of FA on atherosclerotic plaque formation, the lesion area at the aortic sinus was calculated. Compared with control mice, there were obvious atherosclerotic plaques in ApoE^−/−^ mice (Figures 2A–C; *p* < 0.01). However, FA significantly reduced the plaque size by 1.70-fold (*p* < 0.05) compared with the model group. There was no significant difference between FA and simvastatin group. Moreover, FA also stabilized the lesion by increasing the collagen content of fibers (Figure 2A **)**. Meanwhile, H&E staining showed that FA thickened the aortic intima with concomitant thinning of the smooth muscle layer in ApoE^−/−^ mice. The above results indicate that FA treatment could greatly relieve atherosclerosis injury and delay plaque deterioration.

### FA Improved Fatty Liver Injury in ApoE^−/−^ Mice Induced by HFD

**FIGURE 3:**
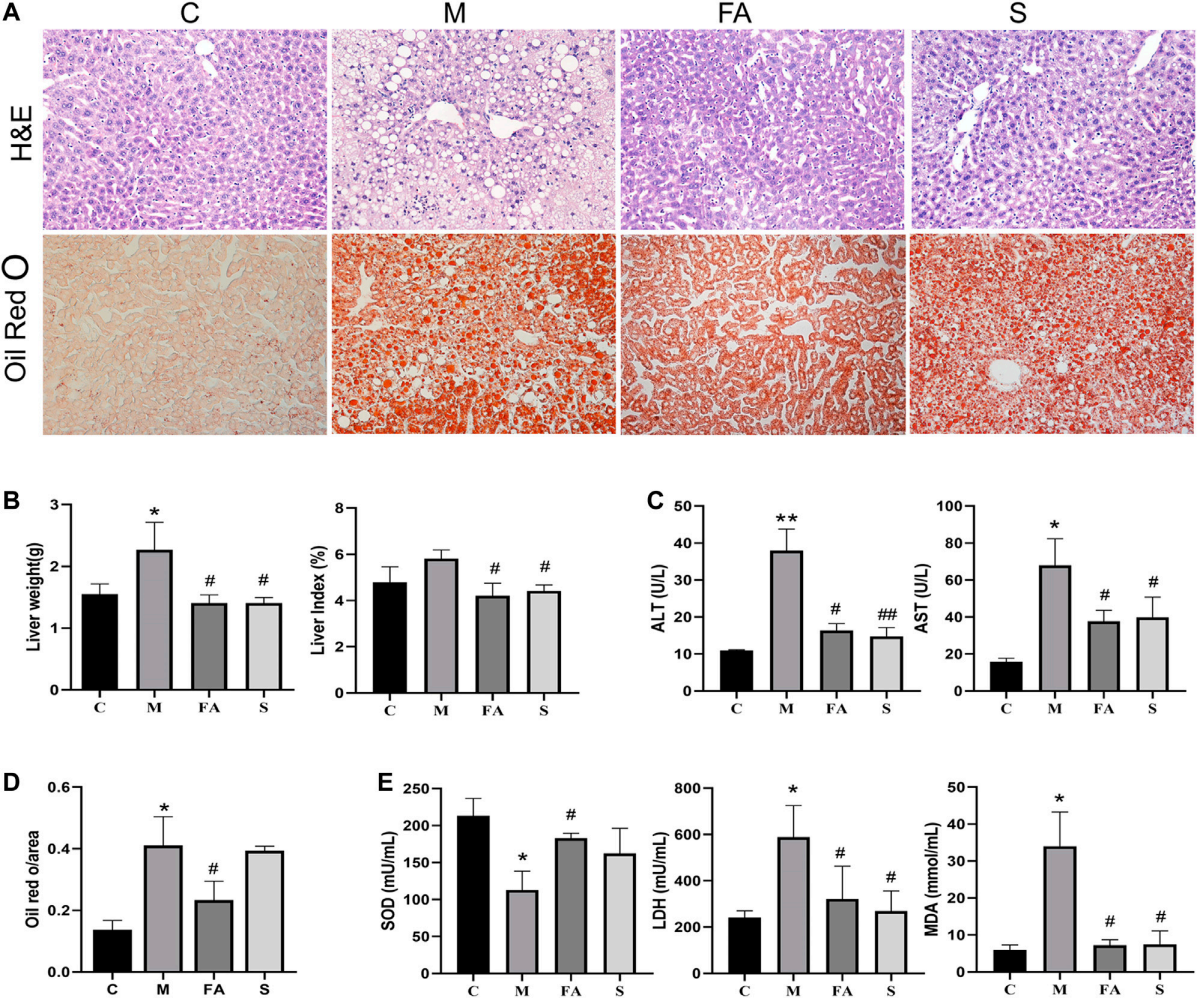
FA improved fatty liver injury in ApoE^−/−^ mice induced by HFD. **(A)** H&E staining of liver paraffin sections, ORO staining of liver sections (200×). **(B)** Liver weight, liver coefficent in various groups (*n* = 4). **(C)** The level of ALT and AST in serum (*n* = 4). **(D)** Quantitative analysis of Oil red O in liver (*n* = 3). **(E)** The change of serum SOD, LDH, MDA in various groups (*n* = 4). *p < 0.05, **p < 0.01; as compared to the control group. ^#^ *p* < 0.05, ^##^ *p* < 0.01; as compared to the model group.

Long term HFD not only accelerates the progression of atherosclerosis, but also induces redundant lipid deposition in the liver. The liver weight and liver index in model mice was much higher than that in control mice. Compared with model mice, FA and simvastatin significantly reduced the liver weight and index (Figure 3B; *p* < 0.01). Lipid content was also reduced by 46.3% after FA treatment compared with that in model mice (Figure 3D; *p* < 0.01). Meanwhile, FA and simvastatin greatly reduced serum AST and ALT activities (Figure 3C; *p* < 0.01). It is well known that oxidative stress, which could also be induced by the HFD, is closely associated with liver damage. Here, we found that the activity of MDA and LDH in model mice was much higher than that in control mice, while SOD was lower (*p* < 0.01). FA significantly reduced the activity of MDA and LDH as well as increased that of SOD (Figure 3E; *p* < 0.05). Overall, these results suggest that FA could relieve fatty liver damage in ApoE^−/−^ mice fed on HFD.

### FA Regulated the Expression of Lipid Metabolites-Related Genes in Mice Liver

**FIGURE 4:**
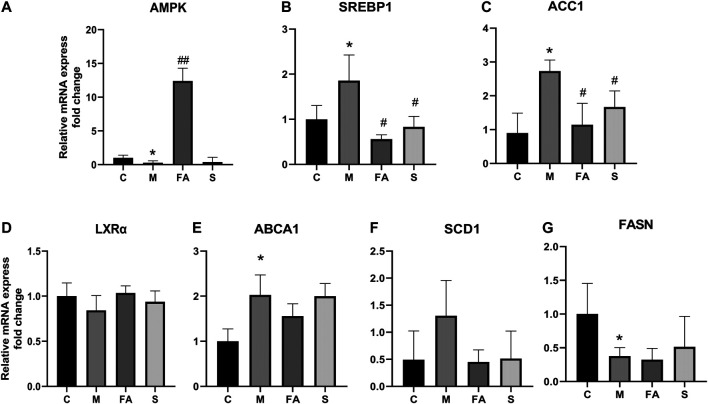
The effect of FA on lipid metabolism related genes in liver **(A)–(G)** Relative mRNA expression of genes related to lipid metabolism in the liver, including LXRα, ABCA1, AMPK, SREBP1, ACC1, SCD1, FASN (*n* = 4). *p < 0.05; as compared to the control group. ^#^ *p* < 0.05, ^##^ *p* < 0.01; as compared to the model group.

**FIGURE 5:**
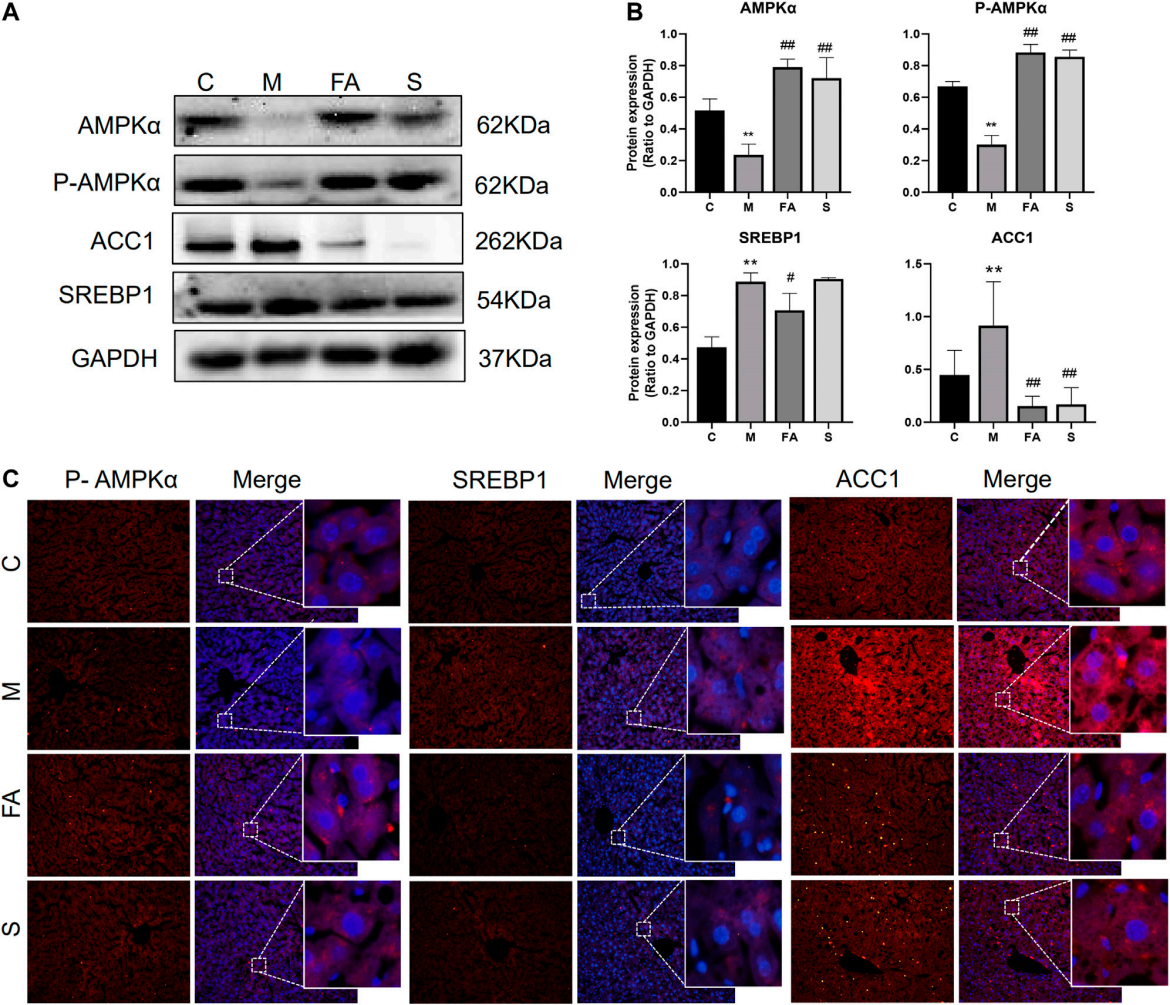
FA regulated the expression of lipid metabolites-related Proteins in HFD-fed ApoE^−/−^ mice **(A), (B)** Protein expression of AMPKα, P-AMPKα, SREBP1, ACC1 in liver (*n* = 3), **(C)** inmmunofluorescence staining of ACC1, SREBP1 in Liver sections (200 ×). *p < 0.05; as compared to the control group. ^#^ *p* < 0.05, ^##^ *p* < 0.01; as compared to the model group.

To explore the underlying mechanism of FA on lipid metabolism, the related genes and protein were evaluated. Firstly, we found that the mRNA expression of SREBP1 (1.86-fold), ABCA1 (2.03-fold), and ACC1 (3.03-fold) was greatly increased in model mice, while the AMPK (0.29-fold) and FASN (0.38-fold) were significantly decreased, compared with those in control mice (Figures 4A–G; *p* < 0.05). FA significantly reduced the mRNA levels of SREBP1 (0.30-fold, *p* < 0.05), ACC1 (0.42-fold, *p* < 0.05) and increased that of AMPK (41.33-fold, *p* < 0.01). However, no obvious difference was observed in the expression of LXRα, ABCA1, FASN or SCD1. Compared with control mice, the phosphorylation of AMPKα was greatly inhibited by 0.45-fold in model mice, while that of ACC1 increased by 1.62-fold (*p* < 0.05). The expression of SREBP1 in the liver was up-regulated 1.87-fold (*p* < 0.05). Compared with model mice, the FA markedly increased protein levels of AMPKα and phosphorylated-AMPKα by approximately 3.43-fold, 2.93-fold and reduced levels of SREBP1, ACC1 protein by 0.79-fold, 0.14-fold, respectively (Figures 5A,B; *p* < 0.05). Moreover, immunofluorescence assays showed a similar trend with western blotting in these target proteins (Figure 5C). Simvastatin also increased the protein level of AMPKα and decreased that of ACC1, which showed no difference compared with FA. Taken together, the above data indicated that FA regulated lipid metabolism by modulation of lipid metabolism-related genes, partly via the AMPKα/SREBP1/ACC1 signaling pathway.

### Effect of FA on Gut Microbiota in ApoE^−/−^ Mice

**FIGURE 6:**
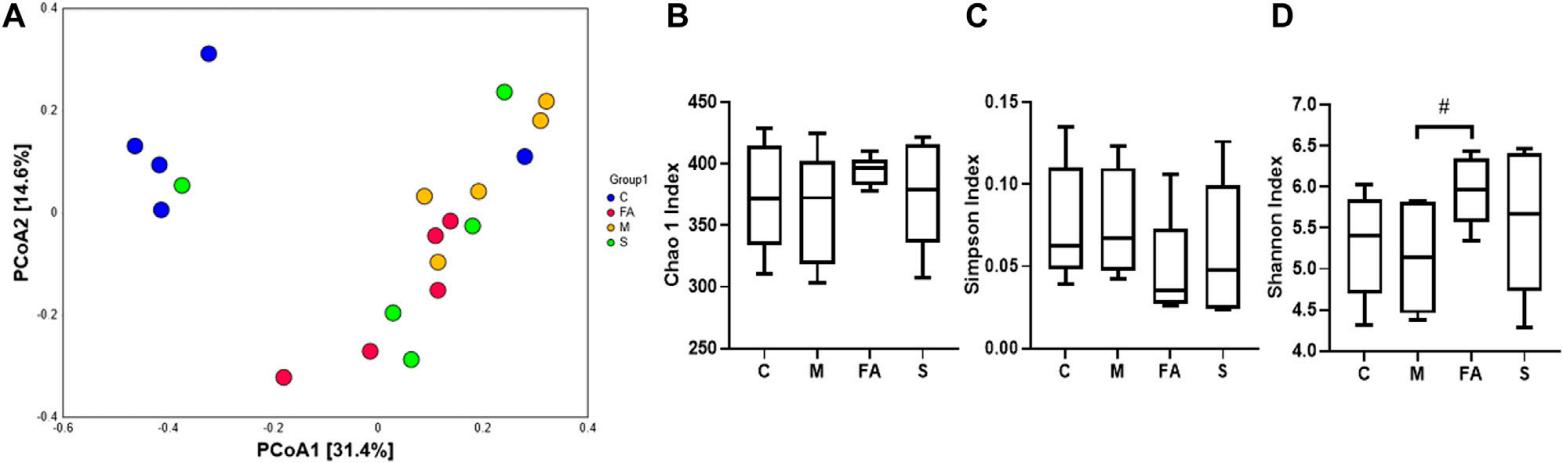
FA increased species diversity of gut microbiota in ApoE^−/−^ mice **(A)** PCoA analysis based on unweighted Unifrac metrics for all samples at the OTU level (*n* = 5). **(B–D)** Diversity of gut microbiota samples from mice, including Chao1, Shannon and Simpson values (*n* = 5). ^#^ *p* < 0.05; as compared to the model group.

The effect of FA on composition of gut microbiota was analyzed by 16S rRNA sequence technology and multivariate analysis. A total of 957951 clean reads were obtained from 20 fecal samples (47897.55 ± 8,437.66 clean reads per sample). After data processing, a total of 623 OTUs (375.10 ±38.65 OTUs per sample) were obtained and then diversity comparison was performed. In our study, there was a marked increase in Shannon values (*p* < 0.05) but no significant difference in Chao1 (*p* = 0.18) and Simpson values (*p* = 0.20) in FA group compared with the model group (Figures 6B–D). As shown in Figure 6A, the unweighted PCoA plot revealed that the cluster from FA group was more similar to model group rather than control group.

**FIGURE 7:**
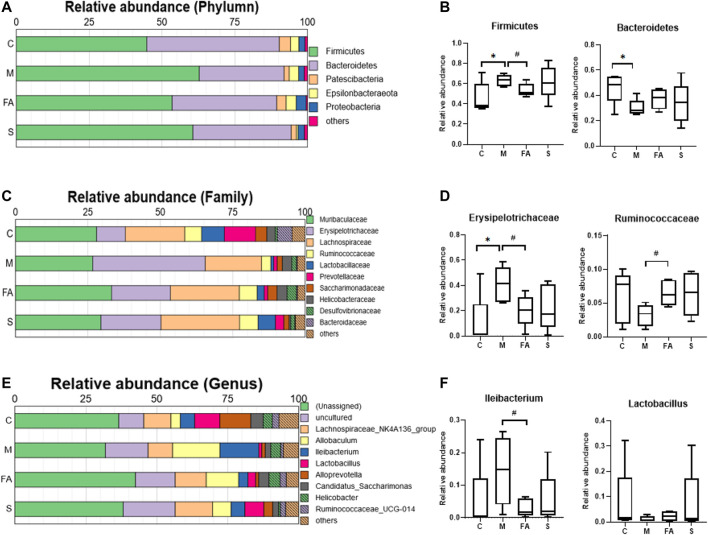
The Effect of FA on gut microbiota composition in ApoE^−/−^ mice **(A)** Relative abundances at phylum level. **(B)** Relative abundances of *Firmicutes* and *Bacteroidetes* (*n* = 5). **(C)** Relative abundances at family level. **(D)** Relative abundances of *Erysipelotrichaceae* and *Ruminococcaceae* (*n* = 5). **(E)** Relative abundances at genus level. **(F)** Relative abundances of *Ileibacterium* and *Lactobacillus* (*n* = 5). *p < 0.05; as compared to the control group. #*p* < 0.05; as compared to the model group.

To assess the role of FA in gut microbiota, the composition of bacteria was analyzed. At the phylum level, *Fimicutes* and *Bacteroidetes* were the dominant bacteria in mice. The HFD resulted in a higher abundance of *Fimicutes* and a lower abundance of *Bacteroidetes* (*p* < 0.05), while FA treatment reduced the abundance of *Fimicutes* (*p* < 0.05) and increased that of *Bacteroidetes* (Figures 7A,B; *p* = 0.17). At the family level, the relative abundance of *Erysipelotrichaceae* was much higher in the model mice than in control mice and FA could obviously reduce it (*p* < 0.05). In addition, the relative abundance of *Ruminococcaceae* was significantly higher in the FA group than that in the model group (Figures 7C,D; *p* < 0.05). At the genus level, the relative abundance of *Ileibacterium* was significantly lower in the FA group than in the model group (*p* <0.05). Conversely, the relative abundance of *Lactobacillus* tended to increase by FA (Figures 7E,F; *p* = 0.28). Simvastatin showed no impact on the gut microbiota composition compared with model group. Briefly, the results indicate that FA could influence the composition and structure of gut microbiota.

### FA Could Modulate the Fecal Metabolites in ApoE^−/−^ Mice

**FIGURE 8:**
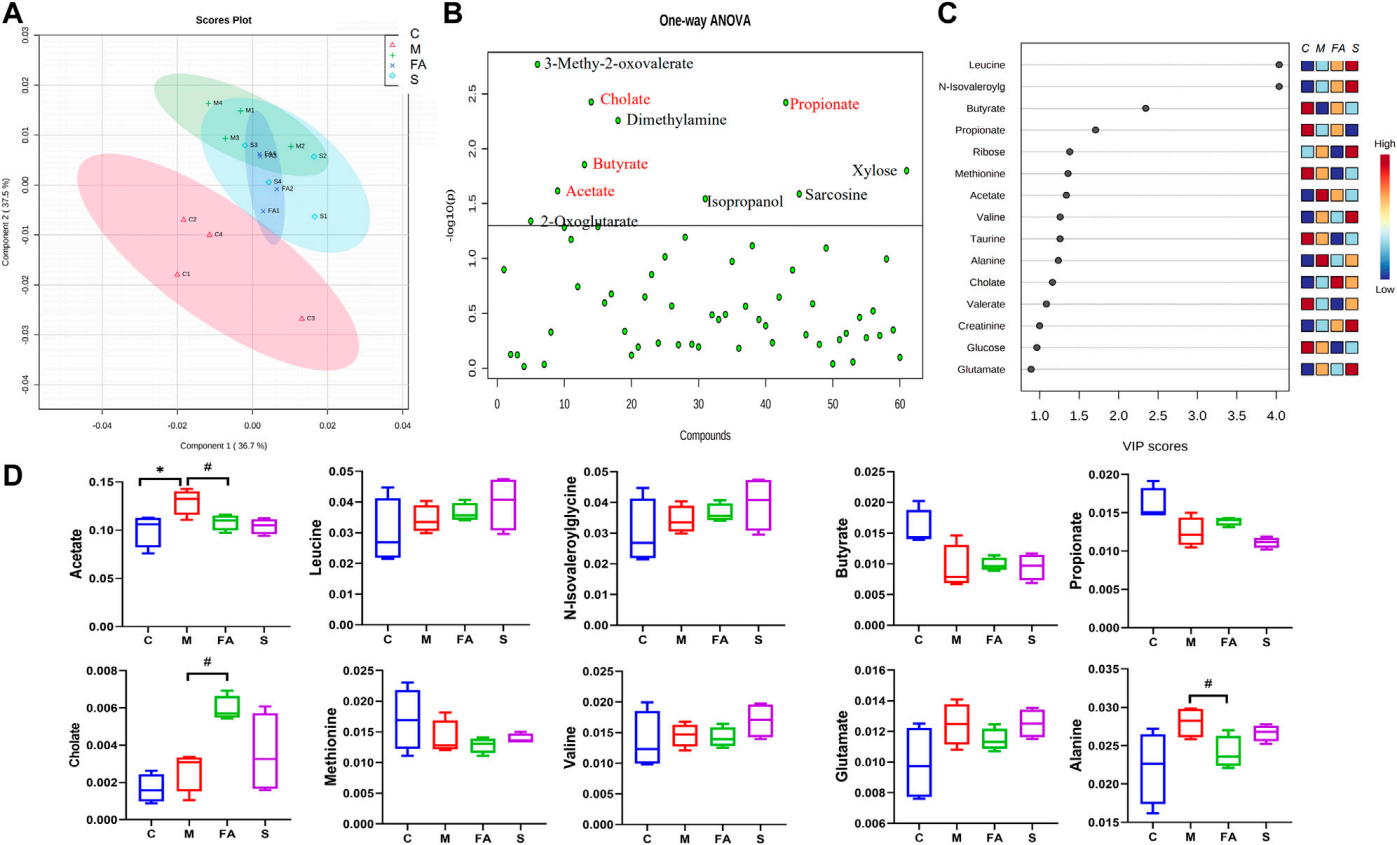
The Effect of FA on gut microbiota metabolite in ApoE^−/−^ mice. **(A)** The PLS-DA Scores plots in per group. **(B)** One-way ANOVA plots of metabolite among groups. The metabolite above the horizontal line had a difference among groups with *p* < 0.05. **(C)** The metabolites with VIP values >1.0 were regarded as important. Red and blue indicated increased and decreased levels, respectively. **(D)** The content of differential metabolites (acetate, leucine, butyrate, propionate, Cholate, valine, Glutamate and alanine) among groups (*n* = 4). ^#^ *p* < 0.05; as compared to the model group.

To evaluate whether FA could affect fecal metabolites, we measured the fecal metabolites by ^1^H NMR. The PLS-DA plot showed an excellent separation of the control and model groups, while FA treatment decreased the distance (Figure 8A). Furthermore, based on the VIP value and statistics analysis, FA could up-regulate cholate and down-regulate acetate and alanine (*p* < 0.05) compared with model group. There was no significant difference in other metabolites such as leucine, butyrate, propionate, and valine (*p* >0.05, Figures 8B–D). Simvastatin showed no regulation in these metabolites compared with model group. Together, the above data suggest that FA treatment modulated the fecal metabolites in ApoE^−/−^ mice.

### Correlation Between Gut Microbiota and Atherosclerosis

**FIGURE 9:**
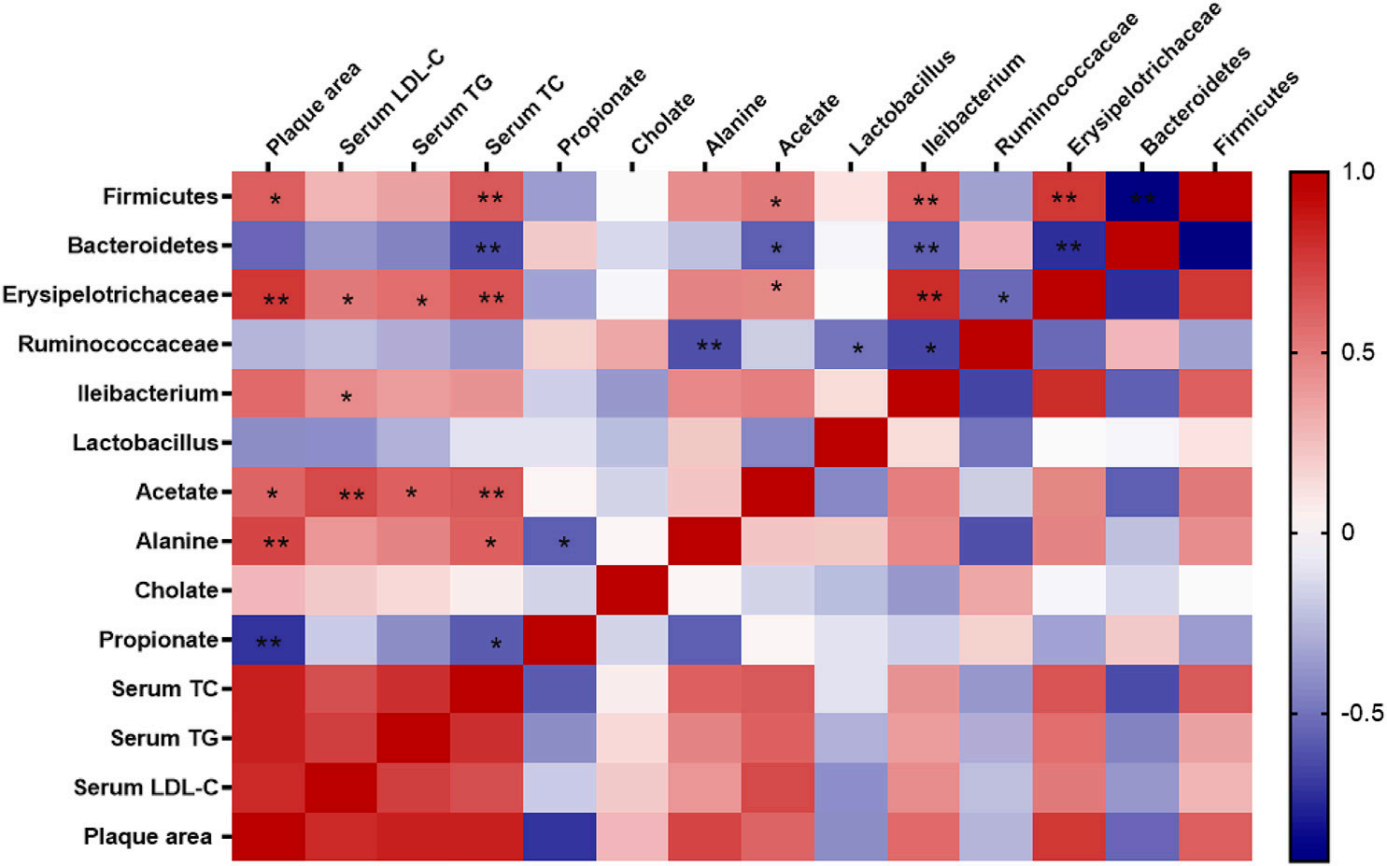
Correlations between gut microbiota and atherosclerosis. Spearman correlation analysis (heatmap) was used to analyze the correlations between gut microbiota and physiological indices including serum levels of lipids and plaque area. The correlation analysis value was represented by colors of grids. Red stood for positive correlation and blue for negative correlation. The deeper red or blue represented higher correlation values. *p < 0.05, **p < 0.01.

Spearman correlation analysis was used to further examine the possible connection between gut microbiota and atherosclerosis. As shown in Figure 9, the relative abundance of *Fimicutes*, *Erysipelotrichaceae*, *Ileibacterium* was positively correlated with atherosclerotic plaque area (*p* < 0.05) and serum lipid level (*p* < 0.05), while *Bacteroidetes* was negatively correlated with serum level TC (*p* < 0.05). Furthermore, the metabolites of gut microbiota including acetate, alanine were positively correlated with the plaque area and serum lipid level (*p* < 0.05), while cholate was not significantly correlated with those. Notably, acetate, alanine was also positively correlated with the relative abundance of *Fimicutes* and *Erysipelotrichaceae*.

## Discussion

**FIGURE 10:**
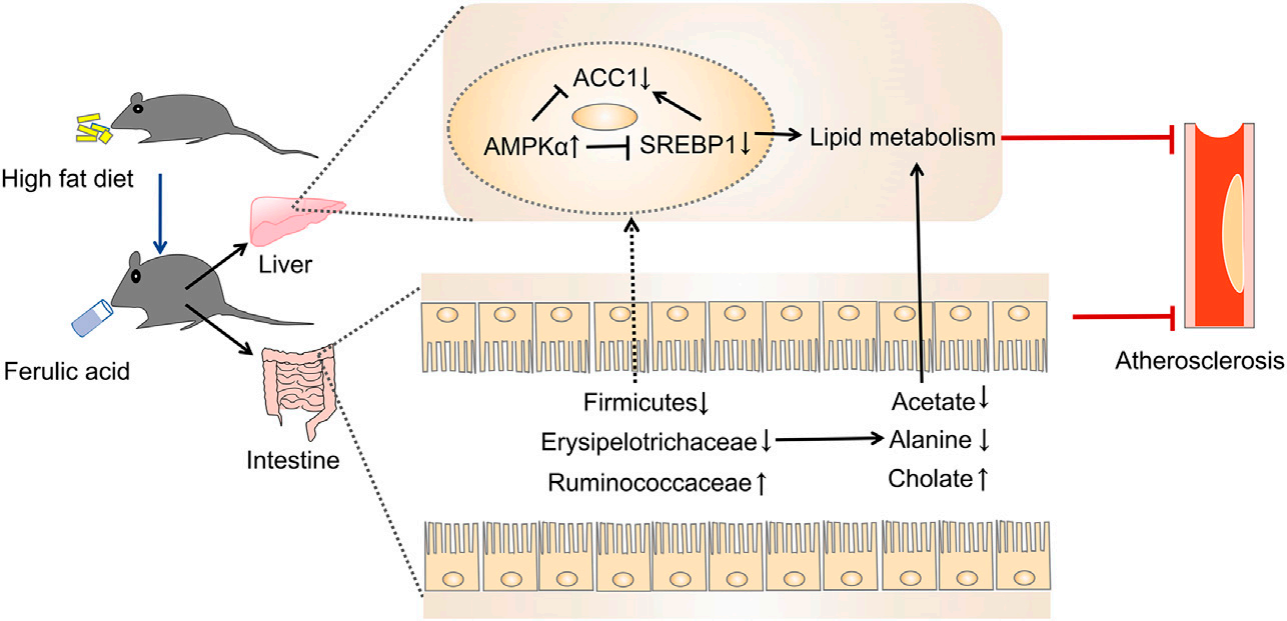
The proposed mechanism for FA reducing atherosclerosis injury in ApoE^−/−^ mice (Huang et al., 2019).

The present study was designed to determine the impact of FA on atherosclerosis in ApoE^−/−^ mice. We found that FA could markedly alleviate atherosclerotic injury in mice. Meanwhile, FA treatment could not only reduce redundant lipid deposition both in the aorta and liver, but also modulate gut microbiota and its metabolites. Moreover, the modulation of FA on the gut microbiota also showed a correlation with atherosclerotic injury. Further study indicated that FA could regulate lipid metabolism, mainly through regulating the lipid metabolism-related genes via the AMPKα/SREBP1/ACC1 pathway. Collectively, FA improved atherosclerosis partly through modulation of gut microbiota and lipid metabolism via AMPKα/SREBP1/ACC1 pathway (Figure 10).

*Angelica sinensis (Oliv.) Diels* and *Ligusticum chuanxiong* has been used in Chinese clinical medicine for more than 2000 years. FA is the main ingredient of both herbs. FA is mainly absorbed in stomach and small intestine after oral administration (Mo et al., 2012). Although FA hardly reaches the cecum, it can be metabolized into three products including cafferic acid, Hydrogenated FA and Demethoxylated FA by the gut microbiota (Zhang et al., 2014). The cafferic acid ameliorates colitis in association with increased *Akkermansia* population in the gut microbiota of mice (Zhang et al., 2016). The effect of cafferic acid on hepatocellular carcinoma may be related to the modulation of the gut microbiota and metabolites (Zhang et al., 2017). FA itself has been identified to modulate the gut microbiota in previous studies (Liu et al., 2019; Ma et al., 2019), which is consistent with our finding here. These studies suggest that FA could possibly influence the gut microbiota through its product, mainly cafferic acid. The effect of FA on atherosclerosis has been reported in the previous study (Chmielowski et al., 2017). In our present study, we found FA not only reduced the plaque area in the aortic sinus comparably with the effect of simvastatin, but also increased the content of fiber collagen to stabilize the plaque. This finding is consistent with another study (Kwon et al., 2010). The results indicated that FA slowed the progression of atherosclerosis in ApoE^−/−^ mice.

Atherosclerosis is a crucial underlying pathology of cardiovascular diseases, and is the leading cause of death worldwide (Liu et al., 2019). Atherosclerosis, which is characterized by atherosclerotic plaque deposits within the aortic intima, develops from lipid disorder and results in vascular stenosis-related diseases, including acute coronary syndrome, heart failure, and stroke. A series of studies has demonstrated that improving lipid metabolism is an effective intervention for atherosclerosis (Ou et al., 2018). Statins have been used as the first-line medicine for patients with atherosclerosis for several decades; however, their side effects, such as myolysis and kidney injury, should not be ignored. Herein, we show that FA treatment significantly decreased the serum level of TC, TG, and LDL-C in mice. The lipid-lowering effect of FA is consistent with previous studies (Kwon et al., 2010; Naowaboot et al., 2016; Ma et al., 2019).

Since HFD always leads to excess cholesterol accumulation in the liver (Feltenberger et al., 2013), the target organ for lipid metabolism, we also evaluated the effects of FA on fatty damage in the liver. We found that FA treatment reversed the increase in liver weight and the liver index. FA also improved liver function by regulation of serum ALT and AST levels. Second, FA treatment significantly suppressed redundant lipid deposition in the liver, which is consistent with previous studies (Ma et al., 2019). Lipid accumulation in hepatocytes causes lipid peroxidation and increases oxidative stress (Chienwichai et al., 2019), which is an important mechanism in liver injury (Li et al., 2015; Dey et al., 2020). The antioxidant effect of FA has also been identified in other studies (Bumrungpert et al., 2018). Herein, we found that FA caused a significant decrease in plasma LDH, and MDA levels and an increase in SOD. MDA is an indicator of lipid peroxidation. Collectively, these results suggest that FA treatment improves the liver injury induced by HFD.

AMPK is composed of a catalytic subunit (α-subunit) and two regulatory subunits (β- and γ-subunits). AMPKα is phosphorylated and activated at its threonine residue (Thr 172) by increasing AMP/ATP ratio and reactive oxygen species (Emerling et al., 2009; Garcia and Shaw, 2017). As a metabolic master switch in the regulation of hepatic lipid homeostasis (Li et al., 2018), AMPK improves lipid metabolism including lipogenesis, lipolysis, lipid transport and oxidation (Ma et al., 2017; Park et al., 2017). Additionally, some studies have reported that AMPK activators suppressed atherosclerotic plaque size by reducing arterial deposition of excess lipids (Vasamsetti et al., 2015; Kimura et al., 2020). Herein, we found that FA treatment significantly increased the phosphorylation of AMPKα. Furthermore, other studies have shown that AMPK not only reduced lipogenesis by regulating SREBP1, ACC (Jung et al., 2012; Park et al., 2017), but it also regulated lipid transport by activating the expression of LXRα and ABCA1 in human macrophages (Kemmerer et al., 2016). We also found that FA significantly down-regulated the expression of SREBP1, ACC1 in atherosclerotic mice. However, there was no significant difference in mRNA level of LXRα or ABCA1. As an important mediator in regulating lipid metabolism, SREBP1 activates the synthesis of fatty acids and triglycerides (Shao and Espenshade, 2012; Zhang et al., 2019). It has been reported that AMPK directly represses the cleavage processing and suppressed the transcription of SREBP1 by phosphorylation (Li et al., 2011). Further, AMPK activation was reported to ameliorate atherosclerosis and hepatic steatosis by inhibiting SREBP activity in the liver of obese LDLR^−/−^ mice (Li et al., 2011; Shao and Espenshade, 2012). It is well known that lipogenic genes including ACC1, FASN, and SCD1 are the target genes of SREBP1 (Horton et al., 2002). In this study, FA has significantly down-regulated the expression of ACC1, which is an essential rate-limiting enzyme in fatty acid metabolism. Inhibition of ACC1 can improve various metabolic diseases including obesity and diabetes (Chen et al., 2019). In brief, we found that FA up-regulated the phosphorylation of AMPKα and down-regulated the expression of SREBP1 and ACC1, which suggested that FA regulates lipid metabolism possibly via the AMPKα/SREBP1/ACC1 pathway in ApoE^−/−^ mice.

Recently, the role of gut microbiota in atherosclerosis has attracted increasing attention. Studies have determined that the impact of gut microbiota on atherosclerosis is closely associated with plasma lipid levels (Lindskog Jonsson et al., 2018). A HFD diet decreased the diversity of gut microbiota (Han et al., 2020), which was reversed by FA treatment in this study. HFD feeding also altered the composition of gut microbiome (Han et al., 2020). At the phylum level, FA significantly decreased the relative abundance of *Firmicutes,* and that of *Bacteroidetes* tended to increase. Lower relative abundance of *Firmicutes* and higher relative abundance of *Bacteroides* are the preventive factors for lipid metabolism (Abdallah Ismail et al., 2011; Lyu et al., 2017). Our results are similar to the previous study (Ma et al., 2019). Analysis at deeper taxonomic level showed that FA decreased the relative abundance of *Erysipelotrichaceae,* which is positively associated with host lipid deposition (Kaakoush, 2015). Furthermore, FA also increased the relative abundance of a health-associated family, *Ruminococcaceae*, which promotes intestinal health and decreases the level of triacylglycerols, phospholipids and cholesteryl esters in mice (Zhang et al., 2020). At the genus level, FA treatment significantly decreased the relative abundance of *Ileibacterium*, which protects mice from adiposity (den Hartigh et al., 2018). Moreover, the relative abundance of *Lactobacillus* tended to increase after mice were treated by FA, which was significantly increased in a previous study (Liu et al., 2019). *Lactobacillus acidophilus* in the intestinal flora regulated lipid metabolism by decreasing cholesterol absorption and regulating reverse cholesterol transport involving PPARα, LXRα, ABCA1 and ABCG1 (Chen et al., 2013; Huang et al., 2014). In addition, *Lactobacillus plantarum NA136* supplementation achieved a lipid-lowering effect via the AMPK pathway, which phosphorylated ACC and suppressed SREBP-1/FAS signaling in HFD-fed mice (Zhao et al., 2019). We also found the relative abundance of *Fimicutes*, *Erysipelotrichaceae*, *Ileibacterium* were positively correlated with atherosclerotic plaque area or serum lipid level here. Overall, the above data indicate that FA regulates lipid levels in atherosclerotic mice possibly through the modulation of gut microbiota composition.

In the present study, we found that FA regulated some metabolites of the gut microbiota. Specifically, cholate was up-regulated, while acetate, alanine down-regulated. Acetate belongs to SCFAs, which affect atherosclerosis after being absorbed by capillaries in the colon (Ohira et al., 2017). Acetate is the major substrates of denovo lipogenesis and cholesterol synthesis, which could inhibit adipose tissue lipolysis in previous study (Yun et al., 2020). Alanine is an amino acid and contributes to lipogenesis by increasing TC and LDL-C in rats (Coqueiro et al., 2019). In addition, cholate may influence atherosclerosis by regulating lipoproteins and the proinflammatory responses (Deeg, 2003). Furthermore, the concentrations of acetate and alanine were positively correlated with atherosclerotic plaque area or serum lipid level in our study, while cholate showed no significant correlation. Given the above, we speculate that FA may regulate lipid metabolism in atherosclerosis mice by modulating the structure and products of the gut microbiota. However, the content of metabolites was only detected in feces and those in serum remained unclear. Therefore, more researches are needed in the future.

## Conclusion

The present study has shown that FA treatment decreases serum lipids and reduces atherosclerotic plaques in ApoE^−/−^ mice. FA also modulates the composition of gut microbiota and fecal metabolites, which is closely related to atherosclerosis. In addition, FA regulates lipid metabolism through activation of the AMPKα/SREBP1/ACC1 pathway in the liver. In brief, we demonstrate that FA could significantly ameliorate atherosclerotic injury, which may be partly by modulating gut microbiota and lipid metabolism via the AMPKα/SREBP1/ACC1 pathway. Nevertheless, the direct link of FA on gut microbiota and atherosclerosis requires further studies.

## Data Availability

The datasets presented in this study can be found in online repositories. The names of the repository/repositories and accession number(s) can be found below: National Center for Biotechnology Information (NCBI) BioProject, https://www.ncbi.nlm.nih.gov/bioproject/, PRJNA678598.

## Abbreviations

ABCA1, ATP-binding cassette transporter A1; ACC1, Acetyl-CoA carboxylase 1; ALT, Alanine aminotransferase; AMPKα, AMP-activated protein kinase α; ApoE^−/−^, ApoE knockout; AST, Aspartate aminotransferase; FA, Ferulic acid; FASN, Fatty acid synthase; HFD, High-fat diet; HDL-C, high density lipoprotein-cholesterol; LDH, lactate dehydrogenase; LDL-C, low density lipoprotein-cholesterol; LXRs, liver X receptors; MDA, malondialdehyde; PCoA, principal coordinates analysis; SOD, superoxide dismutase; SREBPs, sterol-regulatory element binding proteins; TC, total cholesterol; TG, triglycerids
