# Supplementary figures and images for: Ferulic Acid Ameliorates Atherosclerotic Injury by Modulating Gut Microbiota and Lipid Metabolism
Source: Front Pharmacol. 2021 Mar 25;12:621339. doi: 10.3389/fphar.2021.621339 (PMC8026864; doi:10.3389/fphar.2021.621339)

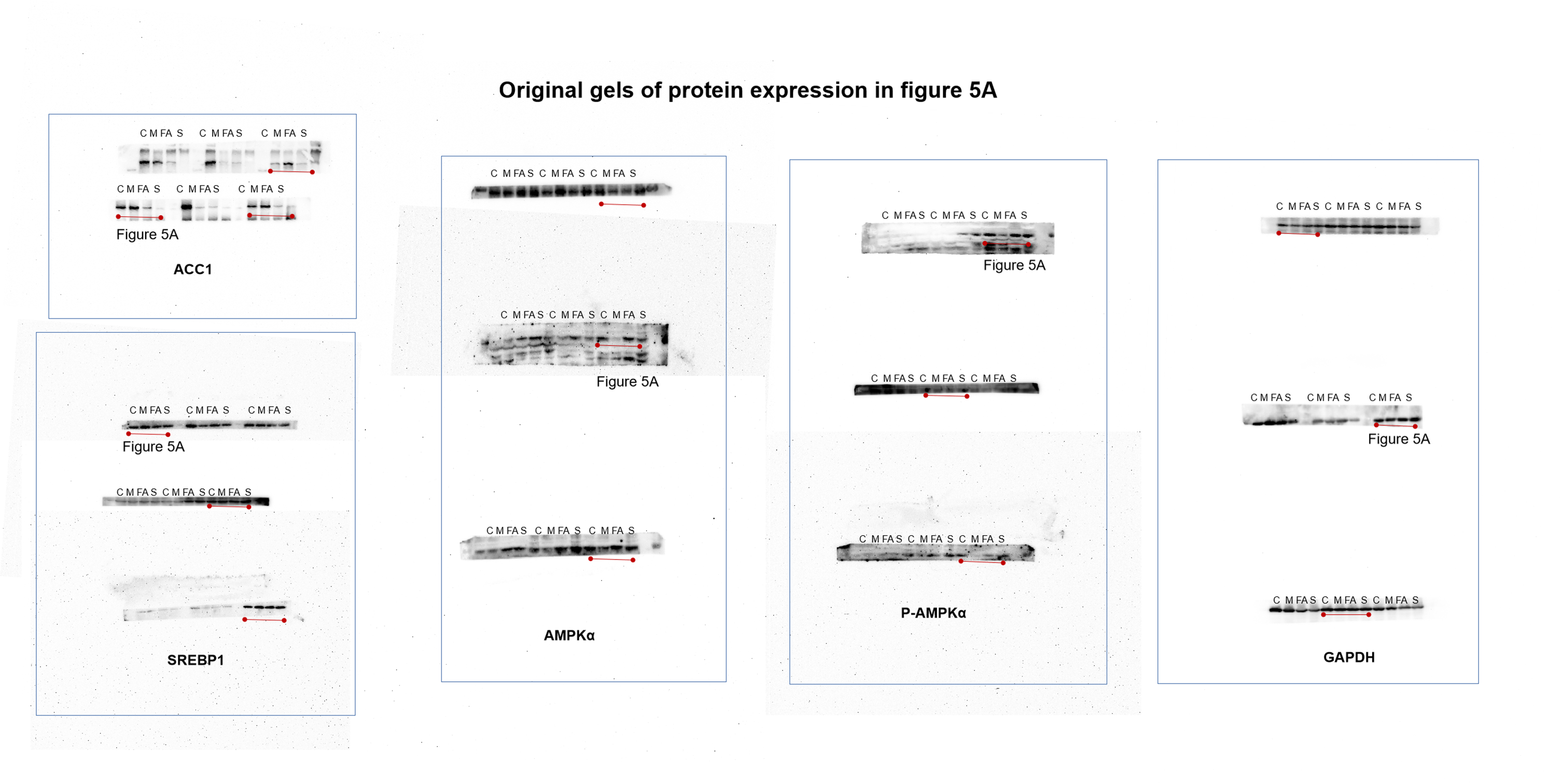

Supplement: Supplementary file 1 [file image1.tif]
